# Plant-based production of highly potent anti-HIV antibodies with engineered posttranslational modifications

**DOI:** 10.1038/s41598-020-63052-1

**Published:** 2020-04-10

**Authors:** Advaita Acarya Singh, Ofentse Pooe, Lusisizwe Kwezi, Therese Lotter-Stark, Stoyan H. Stoychev, Kabamba Alexandra, Isak Gerber, Jinal N. Bhiman, Juan Vorster, Michael Pauly, Larry Zeitlin, Kevin Whaley, Lukas Mach, Herta Steinkellner, Lynn Morris, Tsepo Lebiletsa Tsekoa, Rachel Chikwamba

**Affiliations:** 1 Future Production: Chemicals, Council for Scientific and Industrial Research, Pretoria, South Africa; 2 Discipline of Biochemistry, University of KwaZulu-Natal, Durban, South Africa; 3 Department of Plant and Soil Sciences, University of Pretoria, Pretoria, South Africa; 4 Department of Production Animal Studies, University of Pretoria, Pretoria, South Africa; 5 Mapp Biopharmaceutical, San Diego, California, United States; 6 Centre for HIV and STIs, National Institute for Communicable Diseases of the National Health Laboratory Service (NHLS), Johannesburg, South Africa; 7 Department of Applied Genetics and Cell Biology, University of Natural Resources and Life Sciences, Vienna, Austria; 8 Department of Immunology and Microbiology, The Scripps Research Institute, La Jolla, CA 92037 USA

**Keywords:** Antibody therapy, Recombinant vaccine

## Abstract

Broadly neutralising antibodies (bNAbs) against human immunodeficiency virus type 1 (HIV-1), such as CAP256-VRC26 are being developed for HIV prevention and treatment. These Abs carry a unique but crucial post-translational modification (PTM), namely *O*-sulfated tyrosine in the heavy chain complementarity determining region (CDR) H3 loop. Several studies have demonstrated that plants are suitable hosts for the generation of highly active anti-HIV-1 antibodies with the potential to engineer PTMs. Here we report the expression and characterisation of CAP256-VRC26 bNAbs with posttranslational modifications (PTM). Two variants, CAP256-VRC26 (08 and 09) were expressed in glycoengineered *Nicotiana benthamiana* plants. By *in planta* co-expression of tyrosyl protein sulfotransferase 1, we installed *O*-sulfated tyrosine in CDR H3 of both bNAbs. These exhibited similar structural folding to the mammalian cell produced bNAbs, but non-sulfated versions showed loss of neutralisation breadth and potency. In contrast, tyrosine sulfated versions displayed equivalent neutralising activity to mammalian produced antibodies retaining exceptional potency against some subtype C viruses. Together, the data demonstrate the enormous potential of plant-based systems for multiple posttranslational engineering and production of fully active bNAbs for application in passive immunisation or as an alternative for current HIV/AIDS antiretroviral therapy regimens.

## Introduction

There are an estimated 36.7 million people infected with the Human Immunodeficiency Virus (HIV) worldwide, with about 1 million annual HIV/AIDS-related deaths in 2016^1^. Broadly neutralising antibodies (bNAbs) are attractive alternatives and/or as complements to the current regimens for treatment of HIV infection and are also being evaluated for HIV prevention^2^. Multiple anti-HIV antibodies targeting different viral epitopes exist with varying potency and breadth of neutralisation. A recently reported antibody lineage, CAP256-VRC26 shows promise for further development^3^. CAP256-VRC26 bNAbs target the V1V2 region of the HIV-1 gp120 envelope glycoprotein and some members show exceptional potency against subtype A and C strains^3,4^. The CAP256-VRC26 bNAbs are characterised by several unusual features, one of which is an anionic antigen-binding loop with a protruding *O*-sulfated tyrosine in the CDR H3 loop^3^.

HIV-1 enters the cell by association with the CD4 receptor and critically the CCR5 coreceptor, which has a sulfated tyrosine at the N-terminal end, which is essential for HIV-1 gp120 binding^5^. CDR H3 tyrosine *O*-sulfation is a characteristic feature of antibodies which target the V1V2 region of the HIV-1 gp120 envelope glycoprotein, this emulate the gp120 affinity for the sulfated CCR5^5^. We know that this posttranslational modification (PTM) is crucial for the Ab’s functional activities^6^. The absence of tyrosine sulfation leads to a significant decrease in antigen binding and subsequent loss of function^7^. This typical human type PTM is catalysed by tyrosyl protein sulfotransferases (TPSTs)^8,9^, which makes the expression of such an antibody version restricted to mammalian cell systems which are generally costly and cumbersome^10^. However, alternative and potentially more cost-effective and scalable platforms are available for mass-production of antibodies^10^.

Plants, and in particular *Nicotiana benthamiana*, are well suited for the production of efficacious monoclonal HIV antibodies, such as 2G12, VRC01 and PG9^11–14^. The efficacy of plant-produced versions of some of these antibodies has been tested in animal trials^7^. A remarkable achievement is the generation of Abs with engineered Fc glycosylation resulting in similar or increased Fc receptor binding activities compared to the non-glycoengineered mammalian cell-derived variants^15–17^.

However, the *in planta* generation of bNAbs that need mammalian type PTMs, like tyrosine sulfation, is hampered by the lack of the respective plant enzymatic repertoire and generated recombinant mAbs remain functionally inactive^7^. Notwithstanding, extensive *in planta* engineering approaches allowed for the generation of IgGs (and other proteins) with engineered PTMs^18^. Concurrently, *in planta* CDR H3 tyrosine *O*-sulfation of PG9 and PG16 bNAbs that, like CAP256-VRC26, require this PTM for antigen binding, was reported. This was achieved by the overexpression of human TPST1 (hTPST1) in *N. benthamiana*^14^. Also, the presence of engineered Fc glycans allowed for the production of PG9 and PG16 versions with increased effector functions compared to the mammalian cell-derived variants^14^.

In this report, we demonstrate the expression and characterisation of CAP256-VRC26 bNAbs with engineered PTMs to maintain Ab function. Two versions of CAP256-VRC26 (08, 09) were produced in glycoengineered *N. benthamiana* (ΔXTFT), exhibiting a glycoprofile previously shown *in vitro* to positively impact HIV effector functions of some antibodies^16^. Higher ADCC and ADCVI has been observed *in vitro* only for some HIV bNAbs and no *in vivo* impact has been observed for glycoengineered b12^16^. We further show that by the coexpression of hTPST1, CDR H3 tyrosine sulfation was installed. Our data reveal that PTM engineered CAP256-VRC26 bNAbs exhibited similar structural and functional features compared to HEK293-produced variants, and suggest that plants could be used to mass-produce this antibody for human use.

## Results

### Transient *in planta* coexpression of CAP256-VRC26 bNAbs and hTPST1

Here we used *N. benthamiana* (ΔXTFT), a glycoengineered mutant host that lacks N-glycan residues with a core β1,2-xylose and α1,3-fucose moieties^19^ for transient expression of CAP256-VRC26 bNAbs. Several Potato virus X (PVX) and Tobacco mosaic virus (TMV) vector combinations carrying light and heavy chains, were delivered into plant leaves. Expression levels were measured eight days post-infiltration (d.p.i) by ELISA (Table 1), with the highest production being achieved using the murine IgG heavy chain signal peptide and PVX-mHC + TMV-mLC vector combinations. Expression levels of assembled Abs were 489 and 487 mg.kg^−1^, respectively.

**Table 1:** Determination of vector and signal peptide effects on CAP256-VRC26 bNAb production.

Antibody	Vectors	Production (mg.kg^−1^)
CAP256-VRC26.08	PVX-bHC + TMV-bLC	422
	PVX-mHC + TMV-mLC	489
	TMV-bHC + PVX-bLC	462
	TMV-mHC + PVX-mLC	338
CAP256-VRC26.09	PVX-bHC + TMV-bLC	404
	PVX-mHC + TMV-mLC	487
	TMV-bHC + PVX-bLC	363
	TMV-mHC + PVX-mLC	397

ELISA data of CAP256-VRC26 bNAbs in *N. benthamiana* (ΔXTFT), using combinations of PVX and TMX based expression vectors and murine IgG heavy chain (m) and barley alpha amylase (b) signal peptides.

Note: Data shown above are from a samples size of n = 1.

### MagReSyn^®^ Protein A microsphere-based approach for the one-step protein A purification of CAP256-VRC26 bNAbs

**Figure 1:**
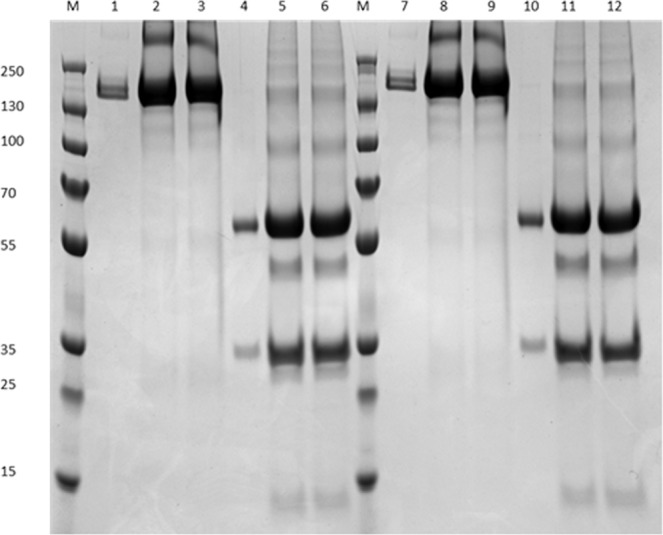
SDS-PAGE analysis of the non-reduced and reduced states of HEK293 and *N. benthamiana* (ΔXTFT)-produced CAP256-VRC26 bNAb. M, Protein Ladder; Lane 1, Non-Reduced HEK293-produced CAP256-VRC26.08; Lane 2, Non-Reduced *N. benthamiana* (ΔXTFT)-produced CAP256-VRC26.08 without hTPST1 coexpression; Lane 3, Non-Reduced *N. benthamiana* (ΔXTFT)-produced CAP256-VRC26.08 with hTPST1 coexpression; Lane 4, Reduced HEK293-produced CAP256-VRC26.08; Lane 5, Reduced *N. benthamiana* (ΔXTFT)-produced CAP256-VRC26.08 without hTPST1 coexpression; Lane 6, Reduced *N. benthamiana* (ΔXTFT)-produced CAP256-VRC26.08 with hTPST1 coexpression; M, Protein Ladder; Lane 7, Non-Reduced HEK293-produced CAP256-VRC26.09; Lane 8, Non-Reduced *N. benthamiana* (ΔXTFT)-produced CAP256-VRC26.09 without hTPST1 coexpression; Lane 9, Non-Reduced *N. benthamiana* (ΔXTFT)-produced CAP256-VRC26.09 with hTPST1 coexpression; Lane 10, Reduced HEK293-produced CAP256-VRC26.09; Lane 11, Reduced *N. benthamiana* (ΔXTFT)-produced CAP256-VRC26.09 without hTPST1 coexpression; Lane 12, Reduced *N. benthamiana* (ΔXTFT)-produced CAP256-VRC26.09 with hTPST1 coexpression.

Magnetic Protein A microspheres were used as a one-step protein A purification method for IgG purification from centrifugally clarified *N. benthamiana* (ΔXTFT) leaf extract which was then analysed on SDS-PAGE (Fig. 1). Under non-reducing conditions IgG1s typically display a single band pattern, ~150 kDa – assembled IgG, whereas, under reducing conditions IgG1s typically display a two-band pattern, ~50 kDa – heavy chain (HC) and ~25 kDa – light chain (LC). The use of MagReSyn^®^ Protein A microspheres resulted in successful purification of CAP256-VRC26 bNAbs from clarified samples. *Nicotiana benthamiana* (ΔXTFT)-produced CAP256-VRC26 bNAb eluents display a similar protein banding pattern to their HEK293-produced counterparts. A prominent signal at position 150 kDa was obtained under non-reducing conditions, corresponding to the size of an assembled IgG. Under reducing conditions, two prominent signals at position 55 kDa and 25 kDa were obtained, corresponding to the size of IgG HC and LC, respectively (Fig. 1). In addition, under reducing condition, there were additional bands at position ~10 kDa and ~40 kDa (Fig. 1, lane 5, 6 and 11, 12) *N. benthamiana* (ΔXTFT)-produced CAP256-VRC26 bNAb eluents; these correspond to proteolytic degradation fragments of the IgGs heavy chain as determined by liquid chromatography-tandem mass spectrometry (LC-MS/MS) and liquid chromatography mass spectrometry (LC-MS) (Supplementary Fig. S4 and S8). Heavy chain proteolytic degradation fragments were also observed between 45–48 kDa, with the lighter fragment being undetected.

### *In planta* sulfation of the CAP256-VRC26 bNAbs requires the coexpression of hTPST1

Sulfation is important for increased antigen-binding affinity and increased neutralisation potency of several mAbs^14,20^. The sulfation state of tryptic CDR H3 peptides of the *N. benthamiana* (ΔXTFT)-produced CAP256-VRC26 bNAbs with hTPST1 co-expression was comparatively analysed against HEK293-produced CAP256-VRC26 bNAbs. Two potential tyrosine sulfation sites exist within the CAP256-VRC26 CDR H3 region (TALYFCVKDQREDECEEWWSD**YY**DFGR). Tyrosine sulfation states and abundance were determined using LC-MS/MS (Table 2). Singly and doubly sulfated species were observed for both HEK293 (Table 2), and *N. benthamiana* (ΔXTFT) (Table 2) produced CAP256-VRC26 bNAbs through LC-MS. However, a lower sulfotyrosine abundance was observed in *N. benthamiana* (ΔXTFT)-produced CAP256-VRC26 bNAbs. Two tyrosine (Tyr112 and Tyr113) sulfation sites were identified in all HEK293-produced CAP256-VRC26 bNAbs; with a sulfation abundance of 90.12% and 88.3% for CAP256-VRC26.08 and CAP256-VRC26.09, respectively. *N. benthamiana* (ΔXTFT)-produced CAP256-VRC26.08 and CAP256-VRC26.09 had sulfation abundances of 60.07% and 63.81%, respectively.

**Table 2:** Tyrosine sulfated species abundance within CAP256-VRC26 bNAbs as deduced by Intact LC-MS. Mono- and Di-sulfated species percentage of the CAP256-VRC26 bNAbs were derived from the deconvoluted mass spectra of the respective HEK293 and *N*.

Monoclonal antibody	Non-sulfated Tyr species (%)	Mono-sulfated Tyr species (%)	Di-sulfated Tyr species (%)
HEK293 produced CAP256-VRC26.08	9.88	38.01	52.11
HEK293 produced CAP256-VRC26.09	11.7	52.75	35.55
*N. benthamiana* produced CAP256-VRC26.08	39.93	39.43	20.64
*N. benthamiana* produced CAP256-VRC26.09	36.19	36.4	27.41

*benthamiana* (ΔXTFT)-produced CAP256-VRC26 bNAb (Supplementary Fig. S9).

### ΔXTFT produced CAP256-VRC26 displayed equivalent proportions of *N*-linked glycoforms and non-glycosylated forms

Although not entirely clear for HIV antibodies it is well known that IgG Fc glycosylation can significantly impact antibody effector functions like antibody-dependent cell-mediated cytotoxicity (ADCC) and antibody-dependent, cell-mediated virus inactivation (ADCVI)^21–23^. The theoretical molecular weight of the unglycosylated CAP256-VRC26.08 light and heavy chains is 22852.53 Da and 52565.25 Da, respectively, with the unglycosylated CAP256-VRC26.09 light and heavy chains being 22779.4 Da and 52620.38 Da, respectively. To obtain Ab versions that lack β1,2-xylose and α1,3-fucose-containing N-glycans residues, and preferentially carry an N-glycosylation profile optimal for effector function (i.e., GnGn structures, nomenclature according to proglycan.com), we used the glycoengineered *N. benthamiana* (ΔXTFT) as an expression host. Indeed, intact LC-MS based glycan analyses of the *N. benthamiana* (ΔXTFT)-produced CAP256-VRC26 (Supplementary Fig. S4 and S8) exhibited a single dominant Fc N-glycan species terminating with GlcNAc residues (GnGn structures, also known as G0) (Table 3). In contrast, HEK293-produced CAP256-VRC26 Fc region showed several glycan species, mainly terminating either with GlcNAc and galactose, respectively (Supplementary Fig. S2 and S6). Virtually all of the glyco-species are core fucosylated (Table 3). A striking difference in glycosylation efficiency of the plant and animal cell-derived Abs was observed. While virtually all Fc’s are glycosylated in HEK293-produced bNAbs, only approx. 50% carried this posttranslational modification on plant-produced Fc’s, consistent with other reports of transient plant-produced Abs^12^. Virtually all *N. benthamiana* (ΔXTFT)-produced (Supplementary Fig. S3 and S7) and HEK293-produced (Supplementary Fig. S1 and S5) CAP256-VRC26 light chain species were N-glycosylated, with *N. benthamiana* (ΔXTFT)-produced light chain species having a single G0 N-glycan (Table 3). In contrast, HEK293-produced light chain species were N-glycosylated at two regions with more complex N-glycans which contain galactose and sialylated groups (Table 3).

**Table 3:** Total *N*-glycosylated species within CAP256-VRC26 bNAbs elucidated through Intact LC-MS. The mass for each peak was determined by BioPharmaView™ software.

Monoclonal antibody subunit	G0 (%)	G0F (%)	G1 (%)	G2 (%)	G2F (%)	G2FS1 (%)	G2NS1 (%)	Total glycosylated species (%)	Total non-glycosylated species (%)
HEK293 produced CAP256-VRC26.08 LC	—	—	—	—	83.73	16.27	—	100	—
HEK293 produced CAP256-VRC26.08 HC	26.24	—	38.68	20.1	2.5	—	—	87.52	12.48
HEK293 produced CAP256-VRC26.09 LC	—	—	—	—	—	25.85	77.07	100	—
HEK293 produced CAP256-VRC26.09 HC	14.23	6.92	29.3	26.15	—	—	—	76.6	23.4
*N. benthamiana* produced CAP256-VRC26.08 LC	95	—	—	—	—	—	—	95	5
*N. benthamiana* produced CAP256-VRC26.08 HC	45	—	—	—	—	—	—	45	55
*N. benthamiana* produced CAP256-VRC26.09 LC	98	—	—	—	—	—	—	98	2
*N. benthamiana* produced CAP256-VRC26.09 HC	50	—	—	—	—	—	—	50	50

*N*-glycosylated species percentage of the CAP256-VRC26 bNAbs were derived from the deconvoluted mass spectra of the respective HEK293 and *N. benthamiana* (ΔXTFT)-produced CAP256-VRC26 bNAb (Supplementary Fig. S1–S8).

### Plant and HEK293 cell produced bNAbs exhibit similar structural features

**Figure 2:**
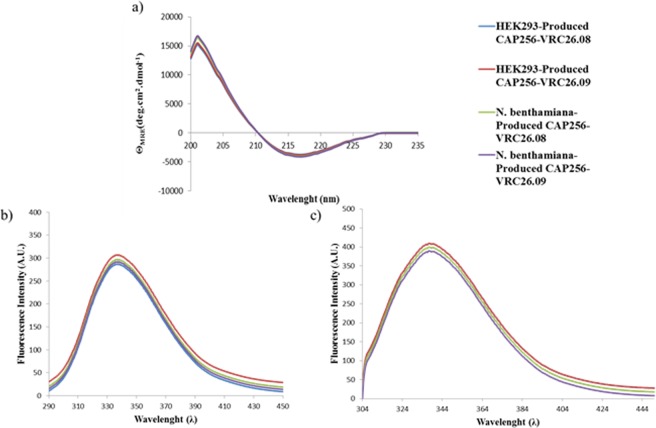
Structural analysis of the CAP256-VRC26 bNAbs. (**a**) Far-UV CD spectra of *N. benthamiana* (ΔXTFT) and HEK293-produced CAP256-VRC26 bNAbs. (**b**) Fluorescence emission spectra of *N. benthamiana* (ΔXTFT) and HEK293-produced CAP256-VRC26 bNAbs excited at 280 nm. (**c**) Fluorescence emission spectra of *N. benthamiana* (ΔXTFT) and HEK293-produced CAP256-VRC26 bNAbs excited at 295 nm.

Preliminary structural analysis was done between the HEK293 and one-step protein A purified *N. benthamiana* (ΔXTFT)-produced CAP256-VRC26 bNAbs, revealing no detectable secondary structural difference between the respective bNAbs. The characteristic minima of the circular dichroism (CD) spectra of the HEK293 and *N. benthamiana* (ΔXTFT)-produced CAP256-VRC26 bNAbs (Fig. 2(a)) is typical of a protein structure with a dominant β-sheet content and low levels of α-helices^24^. The folds of HEK293 and *N. benthamiana* (ΔXTFT)-produced CAP256-VRC26 bNAbs were probed by intrinsic fluorescence (Fig. 2(b,c)). The HC species of CAP256-VRC26.08 and CAP256-VRC26.09 have 11 Trp residues and 17 Tyr residues. The LC species of CAP256-VRC26.08 has 4 Trp residues and 9 Tyr residues and LC species CAP256-VRC26.09 has 5 Trp residues and 7 Tyr residues. All Trp and Tyr residues were distributed similarly throughout CAP256-VRC26.08 and CAP256-VRC26.09. The fluorescence spectral data of both the HEK293 and *N. benthamiana* (ΔXTFT) depict a λ_emm max_ of 337.4 nm. The same λ_emm max_ was exhibited by HEK293 and *N. benthamiana* (ΔXTFT) suggesting that the Trp and Tyr residues are in similar structural environments. This is indicative of similar folds of the CAP256-VRC26 bNAbs from both expression hosts.

### *N. benthamiana* (ΔXTFT) derived bNAbs can neutralise HIV-1 with equivalent efficacy to their HEK293 counterparts *in vitro*

The CAP256-VRC26 lineage is highly potent against HIV-1 subtype A and C strains, with reduced activity against subtype B viruses^3^. Both the one-step protein A purified *N. benthamiana* (ΔXTFT) and HEK293-produced CAP256-VRC26 bNAbs was assessed using the TZM-bl neutralisation assay against a multi-subtype panel of 17 pseudoviruses (Table 4). Representative neutralisation curves of the most sensitive viruses from each subtype are shown in Supplementary Fig. S9. The IC_50_ values of HEK293-produced CAP256-VRC26 bNAbs were similar to those obtained for *N. benthamiana* (ΔXTFT) antibodies co-expressed with hTPST1 neutralising 8 of the 9 subtype C viruses and 3 of the 4 subtype B viruses. A similar pattern was observed for the neutralization of subtype A viruses, where CAP256-VRC26.08 and CAP256-VRC26.09 from both expression systems neutralized 3 and 4 viruses, respectively. Importantly, the exceptional potency against some subtype C and A viruses were maintained by the plant-derived mAbs. However, the non-sulfated *N. benthamiana* (ΔXTFT) antibodies showed loss of both breadth and potency against the panel as indicated by the higher IC_50_ values.

**Table 4:** HIV-1 neutralising activity of CAP256-VRC26 bNAbs produced in *N. benthamiana* (ΔXTFT).

Subtype	Envelope	IC50 (µg/mL)					
		CAP256-VRC26.08			CAP256-VRC26.09		
		HEK produced mAbs	*N. benthamiana* (ΔXTFT) produced mAbs without TPST coexpression	*N. benthamiana* (ΔXTFT) produced mAbs with TPST coexpression	HEK produced mAbs	*N. benthamiana* (ΔXTFT) produced mAbs without TPST coexpression	*N. benthamiana* (ΔXTFT) produced mAbs with TPST coexpression
**C**	**Du172.17**	>50	>50	>50	>50	>50	>50
	**Du156.12**	0.020	0.56	0.020	0.039	0.34	0.030
	**ZM233.6**	0.0032	>50	0.002	0.052	>50	0.015
	**Du422.01**	0.0024	0.22	0.0014	0.047	0.36	0.03
	**ZM197.7**	0.019	0.21	0.034	0.019	0.52	0.015
	**ZM249.1**	0.053	0.47	0.054	0.036	0.42	0.027
	**CAP45.G3**	4.86	18.28	7.68	8.12	46.03	15.73
	**CAP214.15**	1.33	5.16	1.38	0.28	3.97	0.30
**B**	**6535**	>50	>50	>50	>50	>50	>50
	**PVO.4**	8.22	>50	7.83	>50	>50	>50
	**TRO.11**	>50	>50	>50	>50	>50	>50
	**QH0692.42**	>50	>50	>50	>50	>50	>50
**A**	**Q168.a2**	0.14	3.53	0.59	0.22	5.48	0.33
	**Q23.17**	2.78	>50	1.10	4.93	>50	2.34
	**Q842.d12**	>50	>50	>50	5.06	>50	6.32
	**Q461.e2**	1.48	5.71	1.26	0.74	>50	1.00

*Note: Dlata shown above are means of two independent experiments.

## Discussion

This study demonstrates the efficient production of functional anti-HIV bNAbs, CAP256-VRC26 (08 and 09) in *N. benthamiana* (ΔXTFT). At the moment, determining the best signal peptide/vector backbone is more empirical rather than rationally designed. Four different signal peptide/vector backbone combinations were screened to eliminate any low producing combinations. All combinations signal peptide and vector backbone combinations had high (330–490 mg.kg^−1^) absolute levels of recovered mAb.

The presence and functional impact of *O*-sulfated tyrosine in the antigen-binding domains of Abs have so far only been reported for anti-HIV bNAbs that target the V1V2 region of the HIV envelope trimer. Notably, most plants, including *N. benthamiana*, are installed with all the necessary repertoire for carrying out most PTMs, such as transferases for glycosylation, however, previous attempts to find a TPST candidate in the *N. benthamiana* draft genome has been unsuccessful^14^. Indeed Abs that need this modification, remain inactive, as shown in this study and others^7,14^. In the current study, the engineering of tyrosine sulfation within the CDR H3 of the bNAbs was done through co-expression with hTPST1 engineered for post-Golgi targeting^14^. Sulfation was observed in both HEK293 and *N. benthamiana* (ΔXTFT)-produced bNAbs based on a characteristic mass shift observed for tryptic CDR H3 fragments and intact HCs. A mass shift of 79.96 Da per sulfate moiety was observed in both HEK293 and *N. benthamiana* (ΔXTFT)-produced bNAbs, corresponding to reports by Parker and co-workers^25^. Tryptic peptide fragments of CAP256-VRC26 (08 and 09) bNAbs revealed sulfation at two tyrosine residues, Tyr112 and Tyr 113, of which Tyr 112 is essential for the efficacy of the bNAbs^3^. LC-MS revealed mono- and di-sulfated CAP256-VRC26 species in both HEK293 and plant-produced bNAbs. It was, however, observed that there were higher levels of these mono- and di-sulfated CAP256-VRC26 species in the HEK293-produced bNAbs relative to the plant-produced bNAbs. Levels of ~60% sulfation was achieved, similar to what was achieved with PG9^14^. This suggests that the ability of the transiently coexpressed hTPST1 to sulfate tyrosines in the CDR H3 domain might not be as efficient as the native machinery of the HEK293 cells.

Here, we used *N. benthamiana* (ΔXTFT) as an expression platform which allowed the generation of bNAbs carrying virtually exclusively GnGn structures, lacking core xylose and fucose. In contrast, HEK293 cell-derived variants showed four prominent glycan species, the majority decorated with core fucose. Various studies have demonstrated that the lack of fucose on IgG antibodies have superior anti-viral activities due an increased binding to the respective Fcγ-receptor^26,27^. While non-fucosylated anti-HIV mAbs have enhanced FcγR-mediated antiviral activity *in vitro*^14–16^ and show increased ADCC^14^, non-human primate studies are not entirely conclusive. For example fucose free anti-HIV-1 bNAb b12 does not improve protection against SHIV challenge in macaques despite enhanced *in vitro* activities^16^. Further experiments are needed to fully explore the potential of using glycoengineered anti-HIV antibodies *in vivo*.

An important issue with the plant-made CAP256-VRC26 Abs is the incomplete glycosylation of the Fc domain. While Fc domain of HEK293 derived variants are virtually fully occupied by N-glycans only about 50% of plant-produced versions are glycosylated. Incomplete glycosylation of transiently plant-produced Abs has been reported earlier^13,14,19,28^; however, glycosylation levels of 74.3% of G0 glycans were achieved with plant-produced VRC01^13^. It seems that the Fc N-glycosylation site (N297) is inefficiently recognized in some instances by the plant oligosaccharyltransferase complex (OST complex), resulting in under glycosylation of the recombinant glycoproteins. Notwithstanding, *in planta* overexpression of foreign OST subunits is a viable approach to increase *N*‐glycosylation efficiency in plants^12^. Glycosylation of the light chain has been shown to influence the clearance of Ab from blood in pharmacokinetics studies^7^. In contrast to the sialylated glycosylating glycan of the HEK293-made Ab light chain, the glycosylating glycan of the plant-made Ab light chain terminates in a GlcNAc, which has been shown to induce rapid receptor-mediated removal^7^.

Functional activity of CAP256-VRC26 bNAbs was assessed by HIV-1 neutralisation assays *in vitro*. The results demonstrate similar activity of HEK293 cell-derived and sulfated plant-produced Abs. Anti-viral activity was successfully tested against HIV-1 strains (encompassing subtype A, B, and C), confirming maintenance of the broad anti-viral activities of the Abs. In contrast, non-sulfated Ab versions showed a loss of both breadth and potency, emphasizing the importance of *O*-sulfated tyrosine in the CDR H3 domain for functional activity. Interestingly, although the levels of sulfation between HEK293 and plant-expressed antibodies were different (~90% and ~60%, respectively) their neutralization potency was similar. A major problem which is encountered with the plant-based production of protein in *Nicotiana* species, is the proteolytic degradation of some recombinantly produced proteins *in planta*^29,30^. Despite the presence of protease degradation products in the plant produced Abs, similar neutralization potency was seen between the HEK293 cell-derived and sulfated plant-produced Abs samples which contained proteolytic degradation products. This suggests that these degradation products were still functionally active, with cleavage having occurred in a region of the Abs that does not compromise neutralising potency detectable by *in vitro* assay.

Taken together, we demonstrate the efficient *in planta* expression of functionally active CAP256-VRC26 bNAbs with engineered PTMs to optimise efficacy. This study paves the way for further *in vivo* studies to determine the potential of these Abs for treatment of, or passive immunization against HIV/AIDS.

## Materials and Methods

### Mammalian expression of CAP256 bNAbs

Mammalian based expression of the CAP256-VRC26.08 and CAP256-VRC26.09 was done in HEK293 cells at the National Institute for Communicable Diseases (NICD) (Sandringham, JHB, South Africa).

### CAP256-VRC26 cloning

Constructs for the expression of CAP256-VRC26 (08 and 09) were prepared as briefly outlined below. Variable regions sequences were sourced from Doria-Rose and co-workers^3^ and GenBank (AHX01227.1, AHX01239.1, AHX01228.1, AHX01240.1). All variable regions were synthesised and fused to barley α-amylase or murine IgG heavy chain signal peptides and human IgG1 lambda and gamma constant regions. Sixteen vectors were constructed using pICH31180 and pICH21161 MagnICON vectors (ICON Genetics and Nomad Bioscience, Halle, Germany). Various HC/LC 8 vector combinations were infiltrated pair-wise and expression levels were evaluated from 10 g of leaf material (n = 1) using ELISA according to standard procedures (see below).

### *In planta* expression of sulfated CAP256-VRC26 bNAbs

*Agrobacterium*-mediated transient expression system was used for the production of sulfated mAbs. Human TPST1 was co-expressed with the bNAbs as outlined by Loos and co-workers^14^. Syringe agro-infiltration as described by Marillonnet and co-workers^31^ was used. *A. tumefaciens* containing the IgG and hTPST1 constructs were fermented in Luria Broth containing 50 µg.mL^−1^ kanamycin (Kan_50_) and 25 µg.mL^−1^ rifampicin (Rif_25_). *A. tumefaciens* cultures, grown for two days, were pelleted and resuspended in infiltration buffer (10 mM MES, 10 mM MgSO_4_ pH 5.5) and incubated for an hour at 25 °C. CAP256-VRC26.08 and VRC26.09, HC, LC, and hTPST1 infiltration mixtures were co-expressed in the optimal ratio as described by Loos and co-workers^14^. *N. benthamiana* (ΔXTFT)^19^ leaves (4–5 weeks of age) were infiltrated and harvested 8 days post infiltration (d.p.i.). MagnICON based IgG vectors were in *A. tumefaciens* strain LBA4404 (Invitrogen, MA, USA), whereas the hTPST1 vector was in GV3101::pMP90.

### mAb extraction and purification

Briefly, 25 g harvested infiltrated leaves were homogenised in the presence of liquid nitrogen, and proteins were extracted in Phosphate Buffered Saline (1.5 mM KH_2_PO_4_, 8.1 mM NaHPO_4_, 2.7 mM KCl and 140 mM NaCl, pH 7.4) in a 1:2 ratio of leaf material to buffer. bNAbs were purified from centrifugally clarified supernatant. bNAbs were purified using MagReSyn^®^ Protein A microspheres (ReSyn Biosciences, Edenvale, ZA) as per the manufacturer’s instructions.

### SDS-page

IgG samples were separated in the reduced and non-reduced state using a 12% (w/v) polyacrylamide gel which was followed by Coomassie Brilliant Blue staining.

### Elisa

bNAbs were quantified by sandwich ELISA; 96-well plates were coated with either 5 µg/mL goat anti-human lambda LC (bound and free) antibody (L1645, Sigma-Aldrich, St. Louis, USA) or goat anti-human IgG (Fc specific) antibody (I2136, Sigma-Aldrich, St. Louis, USA). A standard concentration range was set using IgG1 (I5029, Sigma-Aldrich, St. Louis, USA). After washing (PBS, pH 7.4 containing 0.1% (w/v) Tween®−20), wells were blocked overnight at 4 °C with 5% fat-free milk in PBS, pH 7.4, followed by washing. Crude *N. benthamiana* (ΔXTFT) extracts were incubated with the respective capture antibody for 2 hours at 37 °C. Post incubation wells were washed and bound IgG’s were detected using either goat α-Human IgG (Fc specific)-peroxidase antibody (A0170, Sigma-Aldrich, St. Louis, USA) and goat α-Human Lambda light chain (bound and free)-peroxidase antibody (A5175, Sigma-Aldrich, St. Louis, USA) which were incubated with sample wells overnight at 4 °C, followed by a wash. 3,3′,5,5′-Tetramethylbenzidine (TMB) substrate solution (Sigma-Aldrich, St. Louis, USA) was used as a peroxidase substrate. Peroxidase-TMB reactions were stopped with 1 M H_2_SO_4_ and readings were taken using a Biotek microplate reader (Winooski, USA) at 450 nm.

### Sulfation analysis of bNAbs

Sulfation analysis was done by in-gel digestion in preparation for LC-MS/MS. Briefly, Coomassie-stained light and heavy chain protein bands were excised from the SDS-PAGE gel and reduced with dithiothreitol (Fermentas, St. Leon-Rot, Germany). Following *S*-alkylation with iodoacetamide (Sigma-Aldrich Co., MO, USA) and digestion with porcine trypsin (Promega, WI, USA), fragments were eluted with 50% (v/v) acetonitrile/5% (v/v) formic acid (Sigma-Aldrich Co., MO, USA). Peptides were desalted and separated using an Acclaim PepMap C18 trap (75 μm × 2 cm) column (Thermo Fischer Scientific, MA, USA) and Acclaim PepMap C18 RSLC column (75 μm × 15 cm) (Thermo Fischer Scientific, MA, USA), respectively using a 4–60% (v/v) gradient of 80% (v/v) acetonitrile/0.1% (v/v) formic acid. Peptides were analysed using an AB Sciex (Miami, USA) 6600 TripleTOF MS, a triple Quadrupole Time of Flight (QTOF) Mass Spectrometer (MS). MS/MS scans were in the m/z range of 100 to 1800 Da. Data analysis was done using Protein Pilot (SCIEX, Canada) and Peaks v6^32^.

### Intact LC-MS analysis of bNAbs

Reduced bNAbs were loaded onto a Jupiter 5 µ C4 300 A (150 ×100 mm) reverse phase chromatography column (Phenomenex, CA, USA) coupled via a switch valve to a 6600 TripleTOF MS, (AB Sciex, Miami, USA) using 80%, 0.1% formic acid and 20%, 80% acetonitrile/0.1% formic acid. Samples were desalted with a linear gradient of 80–70% of 0.1% formic acid and 20–30% of 80% acetonitrile/0.1% formic acid for 0.1 minutes. Samples were eluted into the 6600 TripleTOF MS, using a linear gradient of 70–20%, 0.1% formic acid and 30–80%, 80% acetonitrile/0.1% formic acid for 4.9 minutes. Charge state envelopes were collected within the range 700–2000 Da followed by deconvolution of multiply charged data. Analysis of the MS data was done using AB Sciex Biopharmaview to determine sulfation states and glycosylating glycan species.

### Secondary structure analysis

Far-UV Circular dichroism (CD) spectrum (260–180 nm) measurements of mAb samples in 10 mM Tris-Acetate, pH 7.4 were taken using a 1 mm path length on an Applied Photophysics Chirascan CD spectrometer (Surrey, UK) at 20 °C. Averaged ellipticity values were were converted to mean residue ellipticity (MRE) and corrected for the buffer blank baseline.

### Intrinsic fluorescence analysis

Monoclonal antibodies were selectively excited at 280 and 295 nm using a Shimadzu RF-530K spectrofluorophotometer (Shimadzu Corp., Kyoto, Japan). Fluorescent measurements were taken from 295–500 nm at 20 °C, using a 2 mm quartz cuvette.

### HIV-1 neutralisation assay

The TZM-bl neutralisation assay was performed as described previously^33^. Threefold dilution series of the bNAbs were prepared in Dulbecco’s Modified Eagle’s medium (DMEM) (Sigma-Aldrich Co., MO, USA), with 10% Fetal Bovine Serum (FBS) (Sigma-Aldrich Co., MO, USA) (growth media), at 100 µL per well of a 96-well plate in duplicate. This was followed by the addition of 200 TCID_50_ of HIV-1 pseudovirus/ 50 µL/ well and incubation at 37 °C for an hour. TZM-bl cells at the concentration of 1 × 10^4^ cells/100 µL of growth medium containing 37.5 μg/mL of DEAE dextran per well were added and cultured at 37 °C for 48 hours. The inhibition of HIV-1 infection was determined by measuring the luminescence emitted by the cells using the Infinite F500 plate reader (Tecan, Salzburg, Austria). Titers were calculated as the concentration that caused 50% reduction (IC_50_) of relative light unit (RLU) compared to the virus control (wells with no inhibitor) after the subtraction of the background (wells without both the virus and the inhibitor).

### Ethical statement

This article does not contain any studies with human participants or animals performed by any of the authors.

## Supplementary information

Supplementary information.
