# Supplementary material for: Plant-based production of highly potent anti-HIV antibodies with engineered posttranslational modifications
Source: Sci Rep. 2020 Apr 10;10:6201. doi: 10.1038/s41598-020-63052-1 (PMC7148297; doi:10.1038/s41598-020-63052-1)
Supplement: Supplementary file 1 — Supplementary information. [file 41598_2020_63052_MOESM1_ESM.pdf]

Plant-based production of highly potent anti-HIV antibodies with engineered posttranslational modifications

Advaita Acarya Singh[1,3], Ofentse Pooe[2], Lusisizwe Kwezi[1], Therese Lotter-Stark[4], Stoyan H. Stoychev[1], Kabamba Alexandra[1], Isak Gerber[1], Jinal N. Bhiman[8], Juan Vorster[3], Michael Pauly[5], Larry Zeitlin[5], Kevin Whaley[5], Lukas Mach[7], Herta Steinkellner[7], Lynn Morris[6], Tsepo Lebiletsa Tsekoa[1] and Rachel Chikwamba[1]

Supplementary Information

Supplementary Figure S1. Deconvoluted mass spectra of the HEK293-produced CAP256-VRC26.08 reduced light chain species.

Supplementary Figure S2. Deconvoluted mass spectra of the HEK293-produced CAP256-VRC26.08 reduced heavy chain species

Supplementary Figure S3. Deconvoluted mass spectra of the *N. benthamiana* (ΔXTFT)-produced CAP256-VRC26.08 reduced light chain species.

Supplementary Figure S4. Deconvoluted mass spectra of the *N. benthamiana* (ΔXTFT)-produced CAP256-VRC26.08 reduced heavy chain species.

Supplementary Figure S5. Deconvoluted mass spectra of the HEK293-produced CAP256-VRC26.09 reduced light chain species.

Supplementary Figure S6. Deconvoluted mass spectra of the HEK293-produced CAP256-VRC26.09 reduced heavy chain species.

Supplementary Figure S7. Deconvoluted mass spectra of the *N. benthamiana* (ΔXTFT)-produced CAP256-VRC26.09 reduced light chain species.

Supplementary Figure S8. Deconvoluted mass spectra of the *N. benthamiana* (ΔXTFT)-produced CAP256-VRC26.09 reduced heavy chain species.

Supplementary Figure S9. Representative inhibition curves for the most sensitive virus of the sutypes C, B and A.

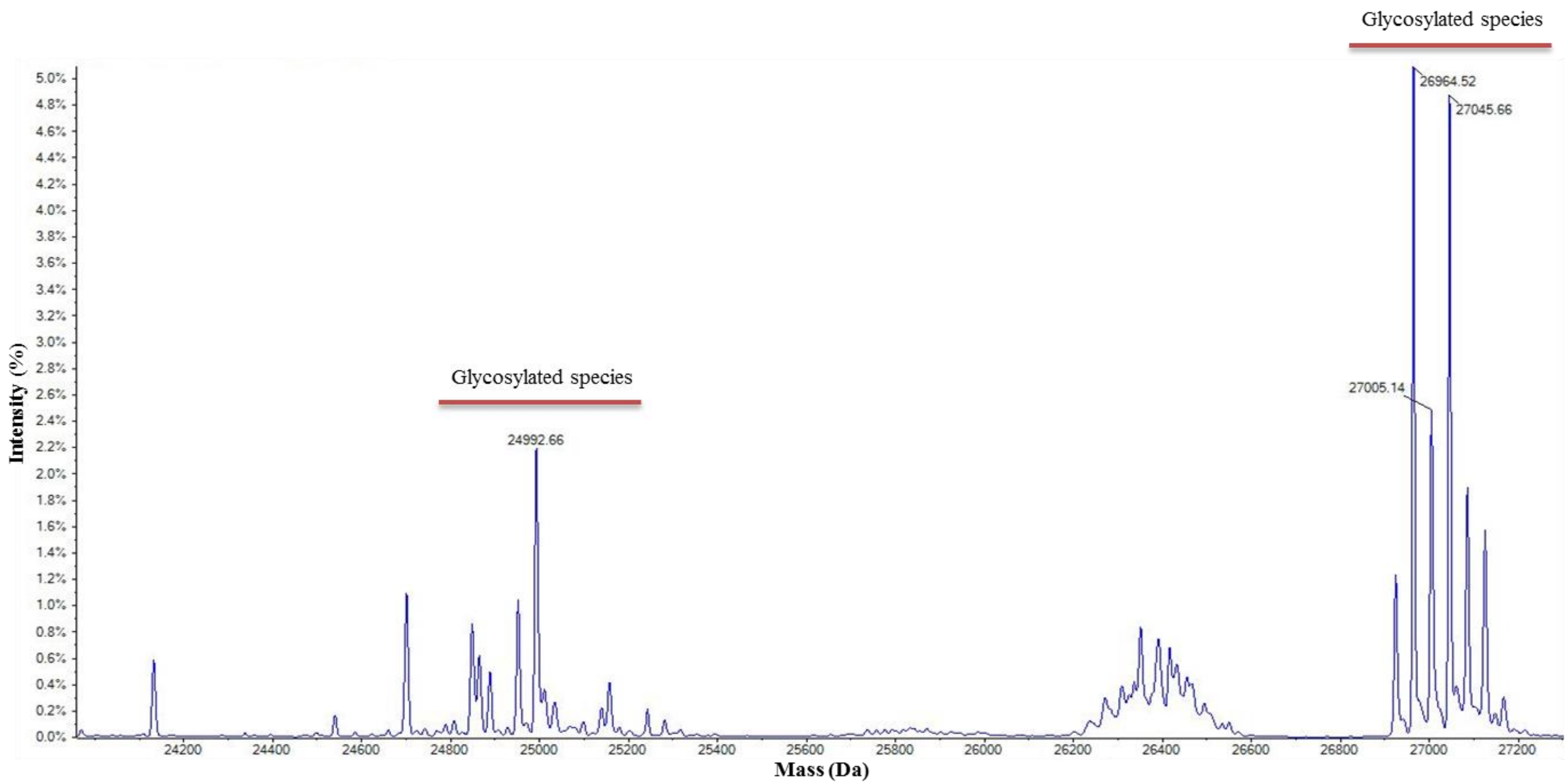

Supplementary Figure S1. Deconvoluted mass spectra of the HEK293-produced CAP256-VRC26.08 reduced light chain species.

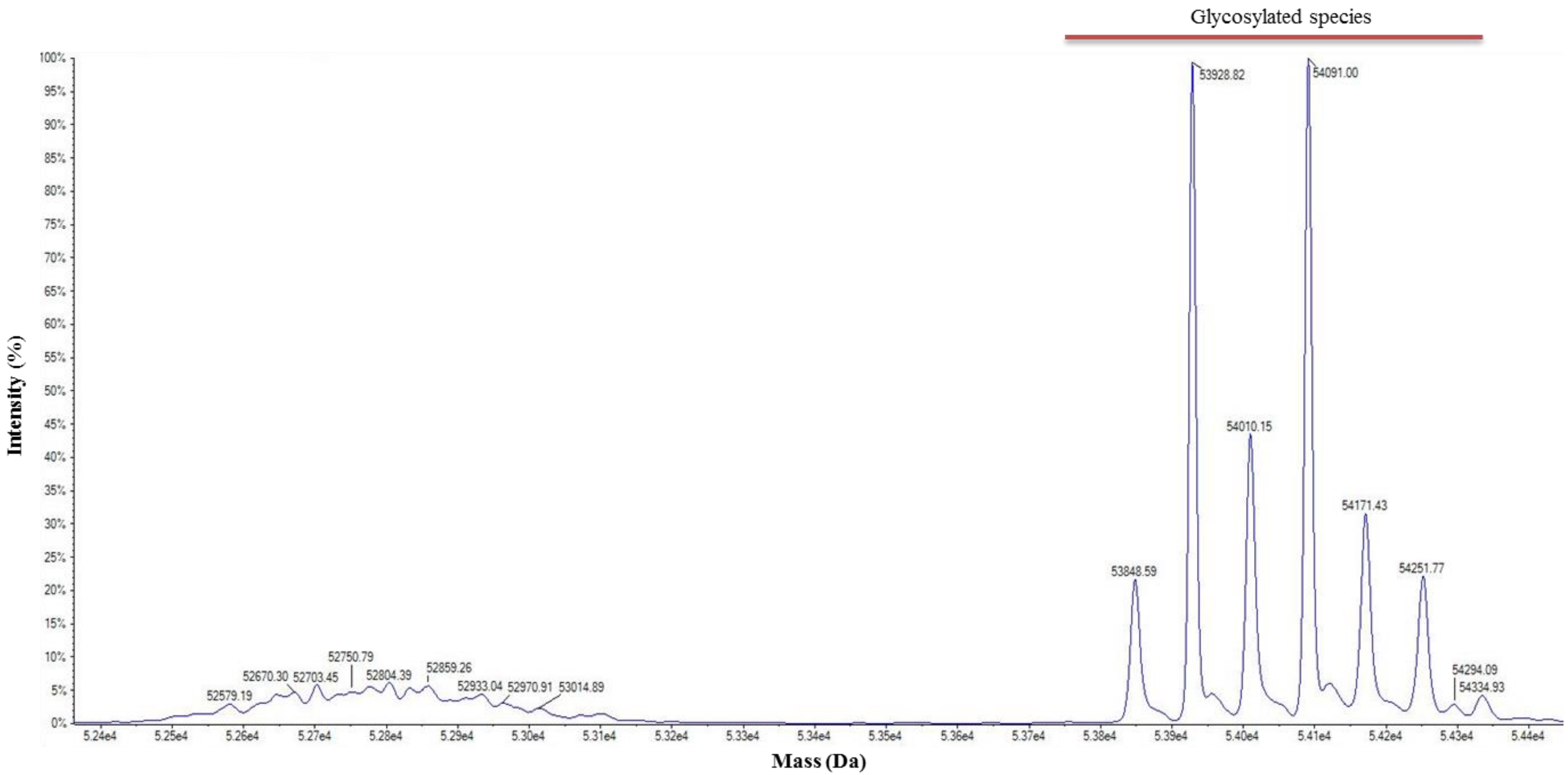

Supplementary Figure S2. Deconvoluted mass spectra of the HEK293-produced CAP256-VRC26.08 reduced heavy chain species.

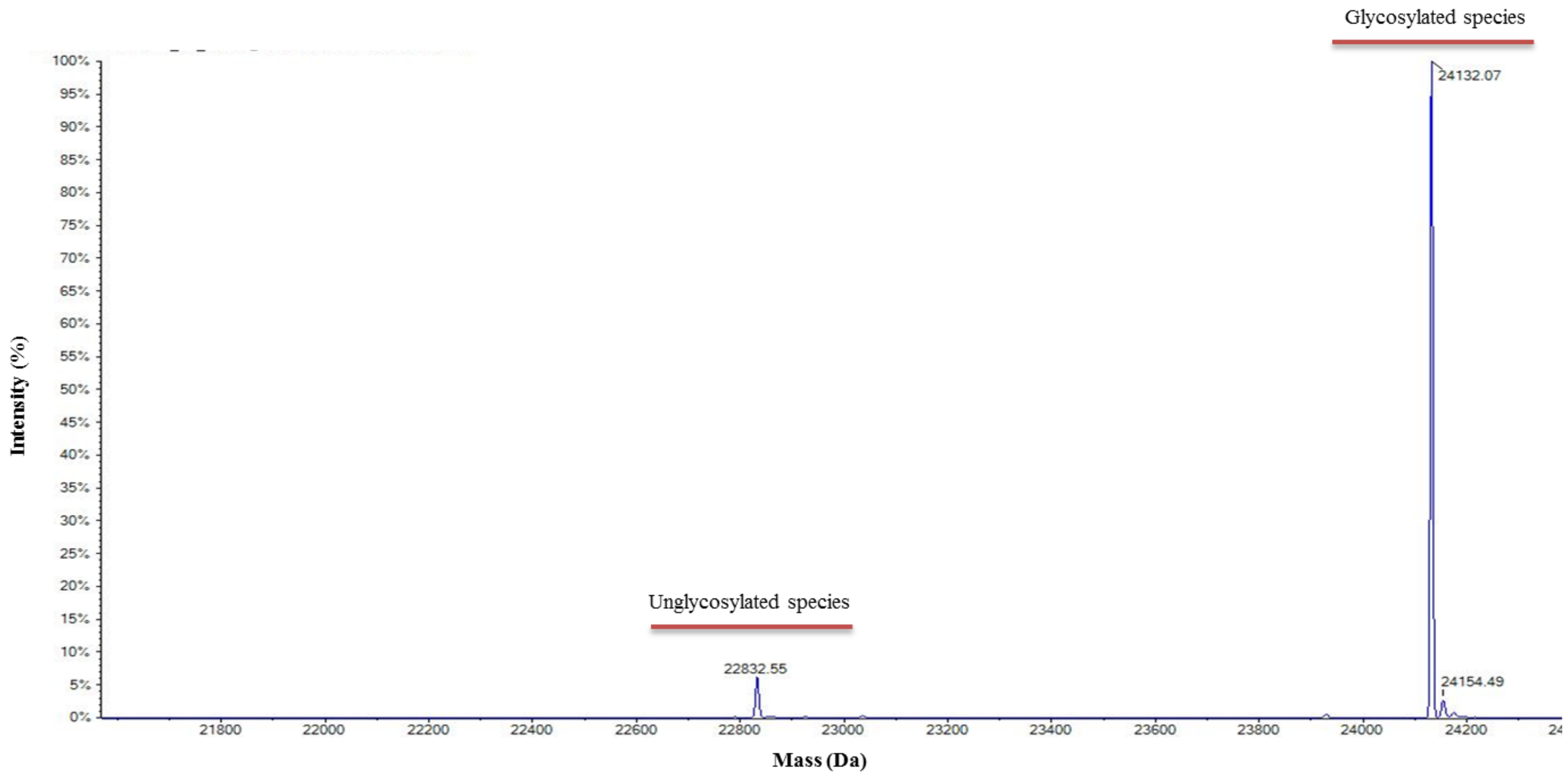

Supplementary Figure S3. Deconvoluted mass spectra of the *N. benthamiana* (ΔXTFT)-produced CAP256-VRC26.08 reduced light chain species.

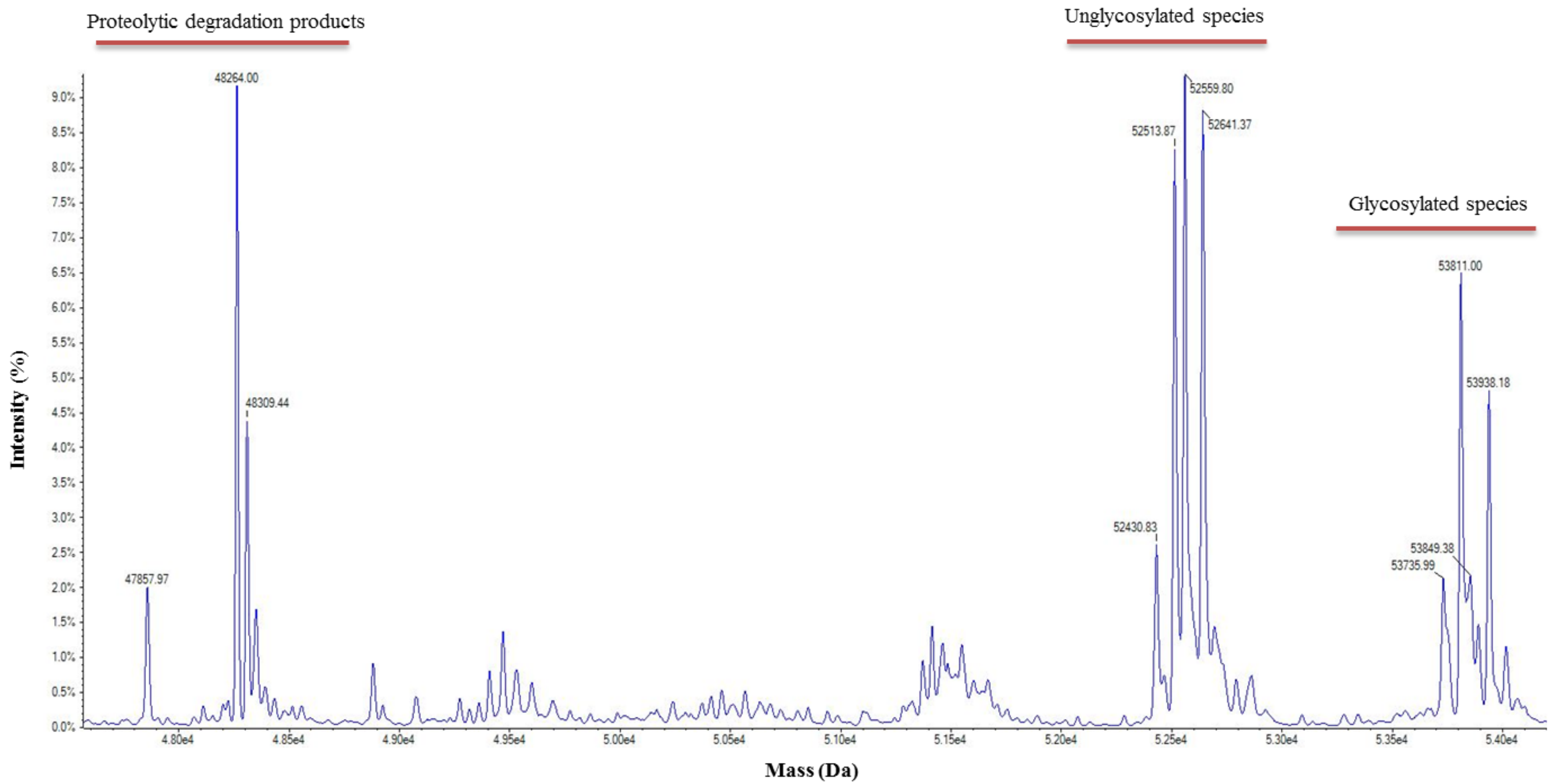

Supplementary Figure S4. Deconvoluted mass spectra of the *N. benthamiana* (ΔXTFT)-produced CAP256-VRC26.08 reduced heavy chain species.

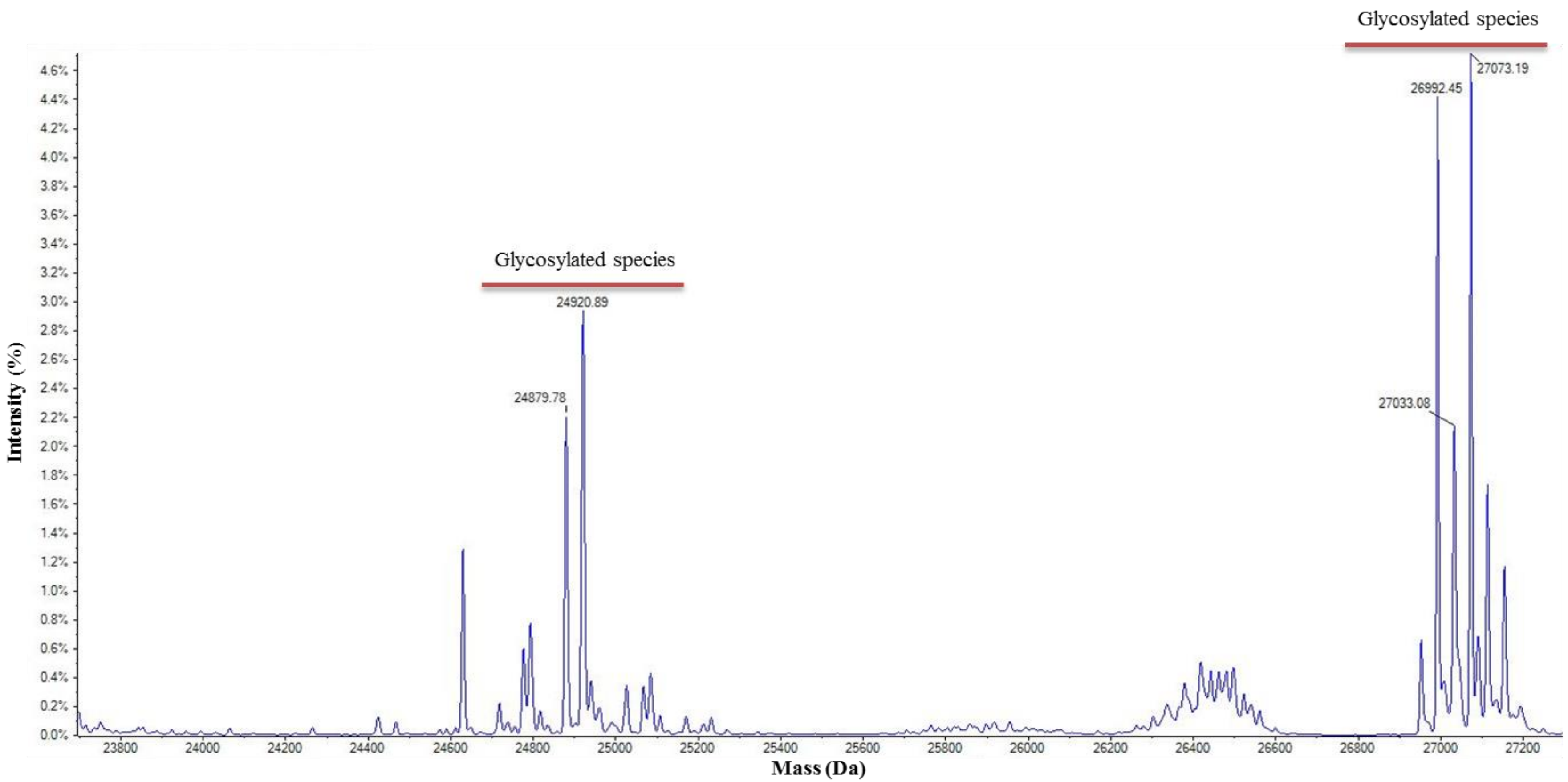

Supplementary Figure S5. Deconvoluted mass spectra of the HEK293-produced CAP256-VRC26.09 reduced light chain species.

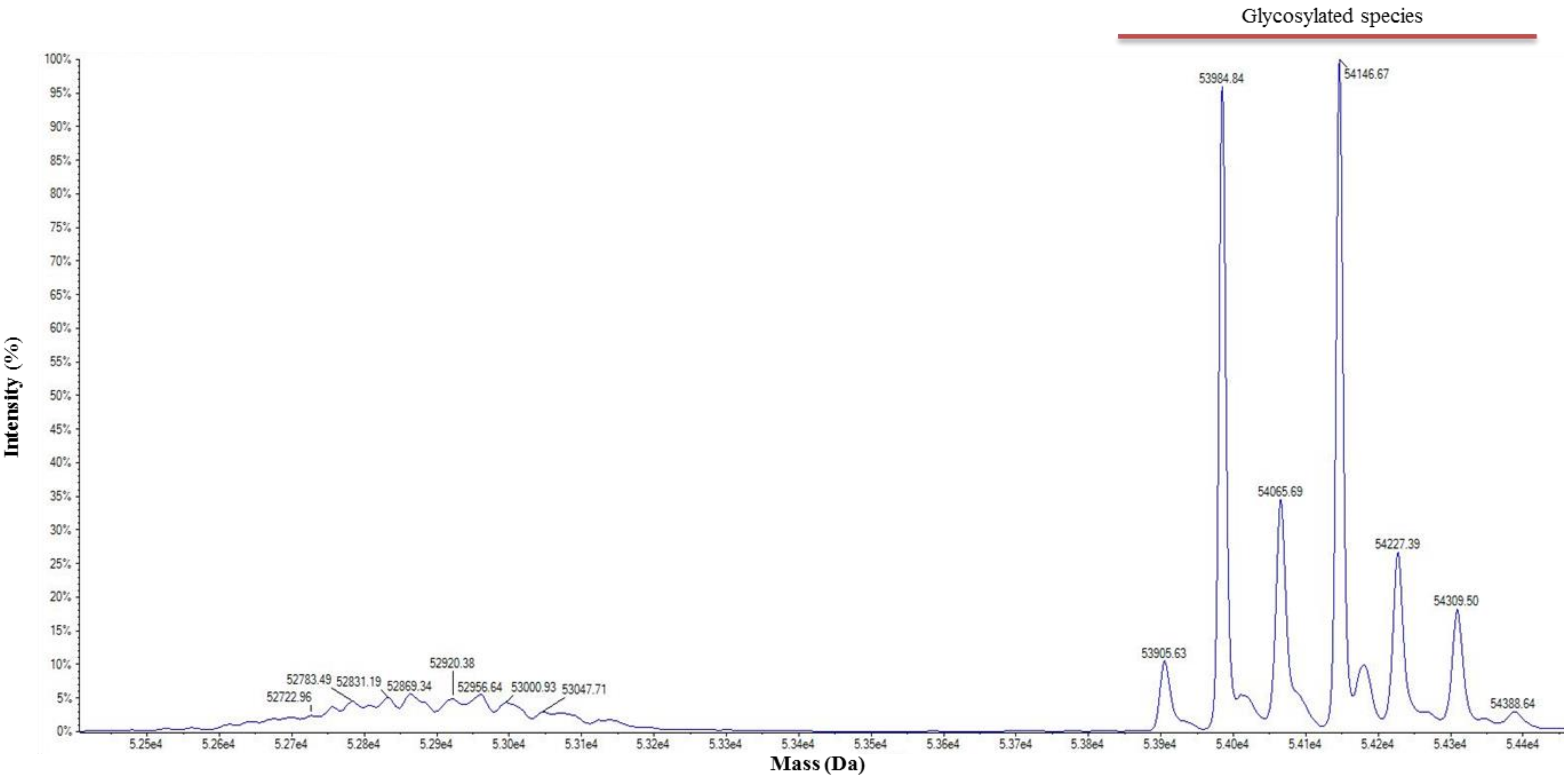

Supplementary Figure S6. Deconvoluted mass spectra of the HEK293-produced CAP256-VRC26.09 reduced heavy chain species.

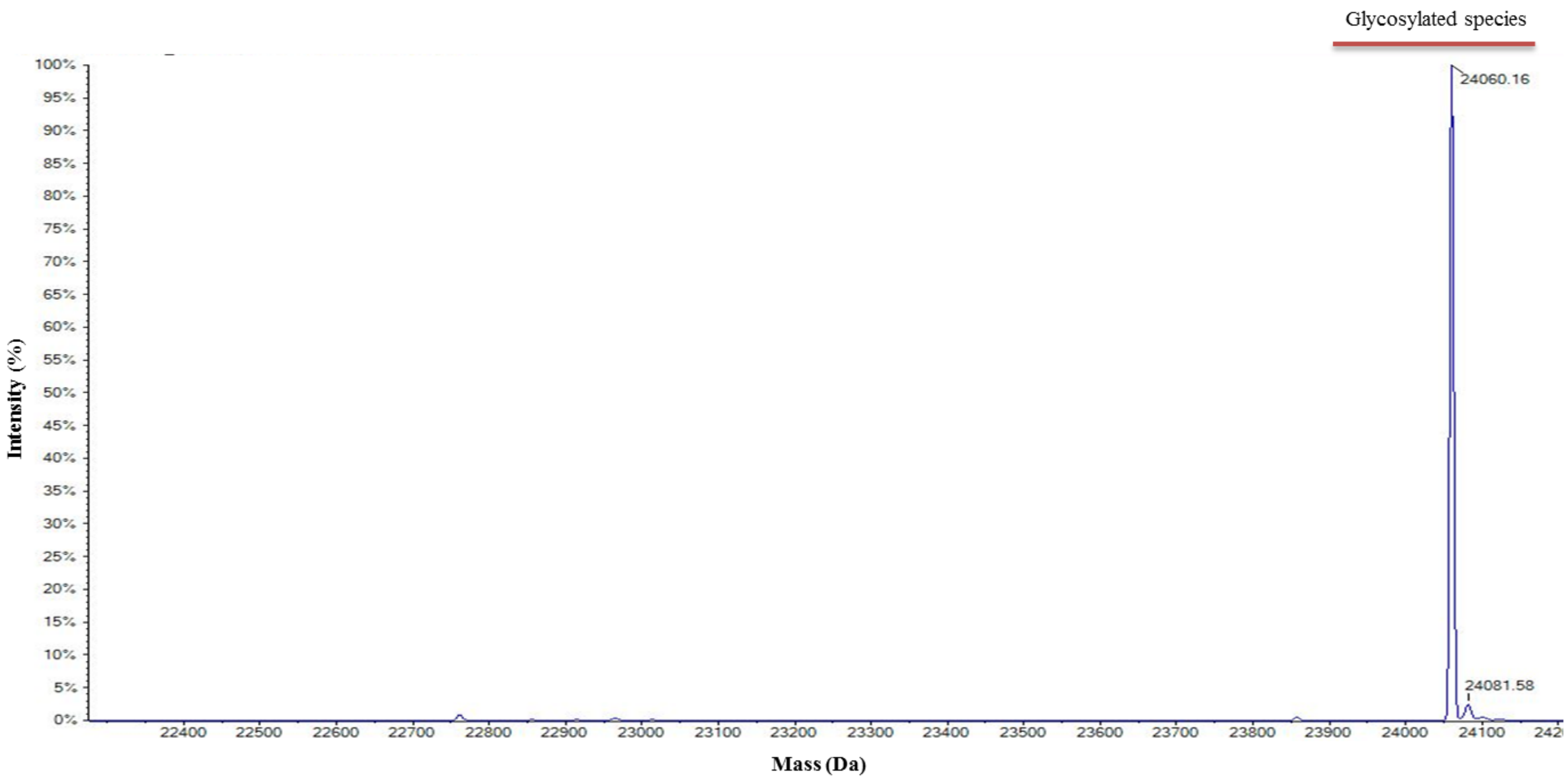

Supplementary Figure S7. Deconvoluted mass spectra of the *N. benthamiana* (ΔXTFT)-produced CAP256-VRC26.09 reduced light chain species.

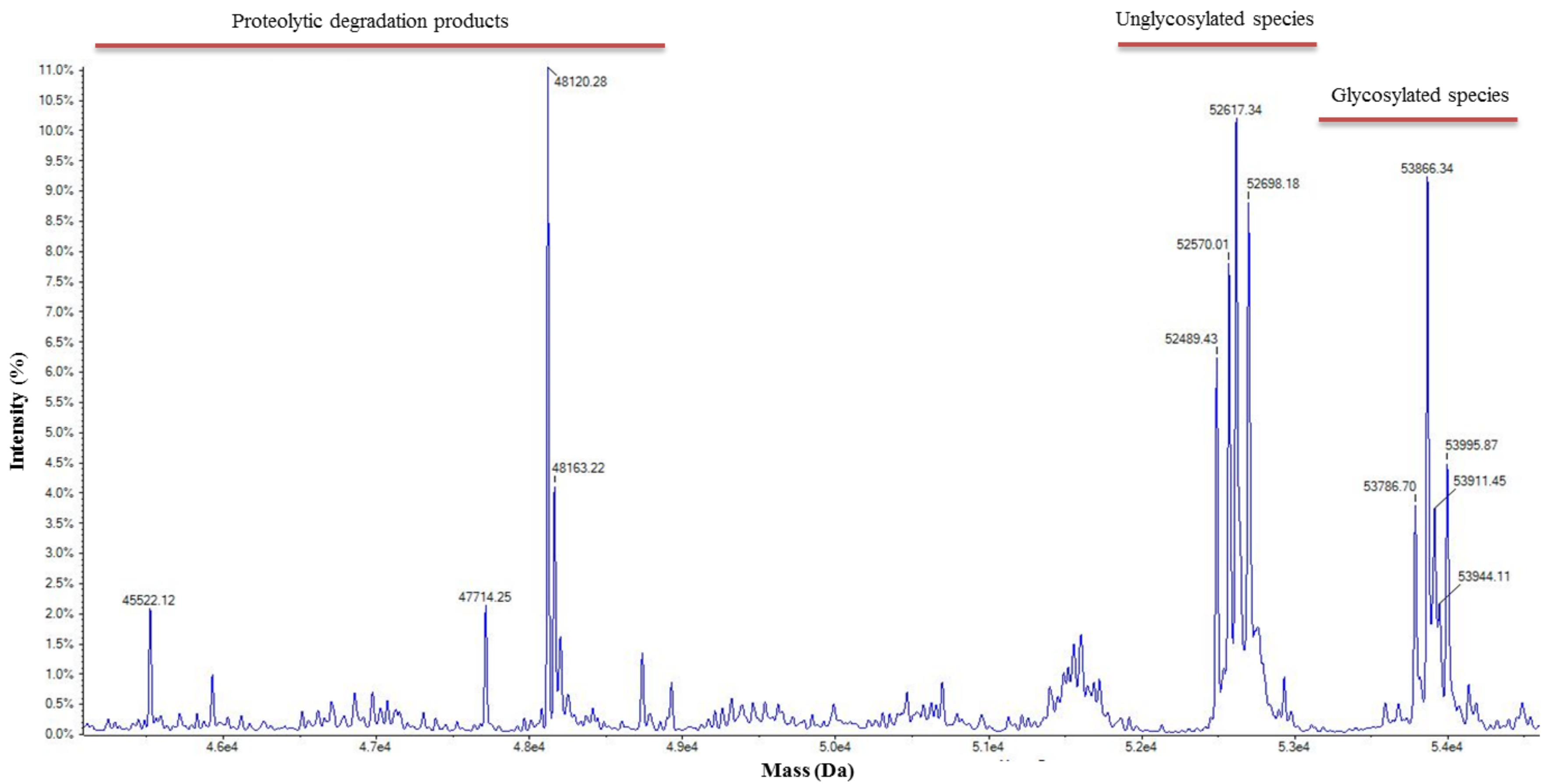

Supplementary Figure S8. Deconvoluted mass spectra of the *N. benthamiana* (ΔXTFT)-produced CAP256-VRC26.09 reduced heavy chain species.

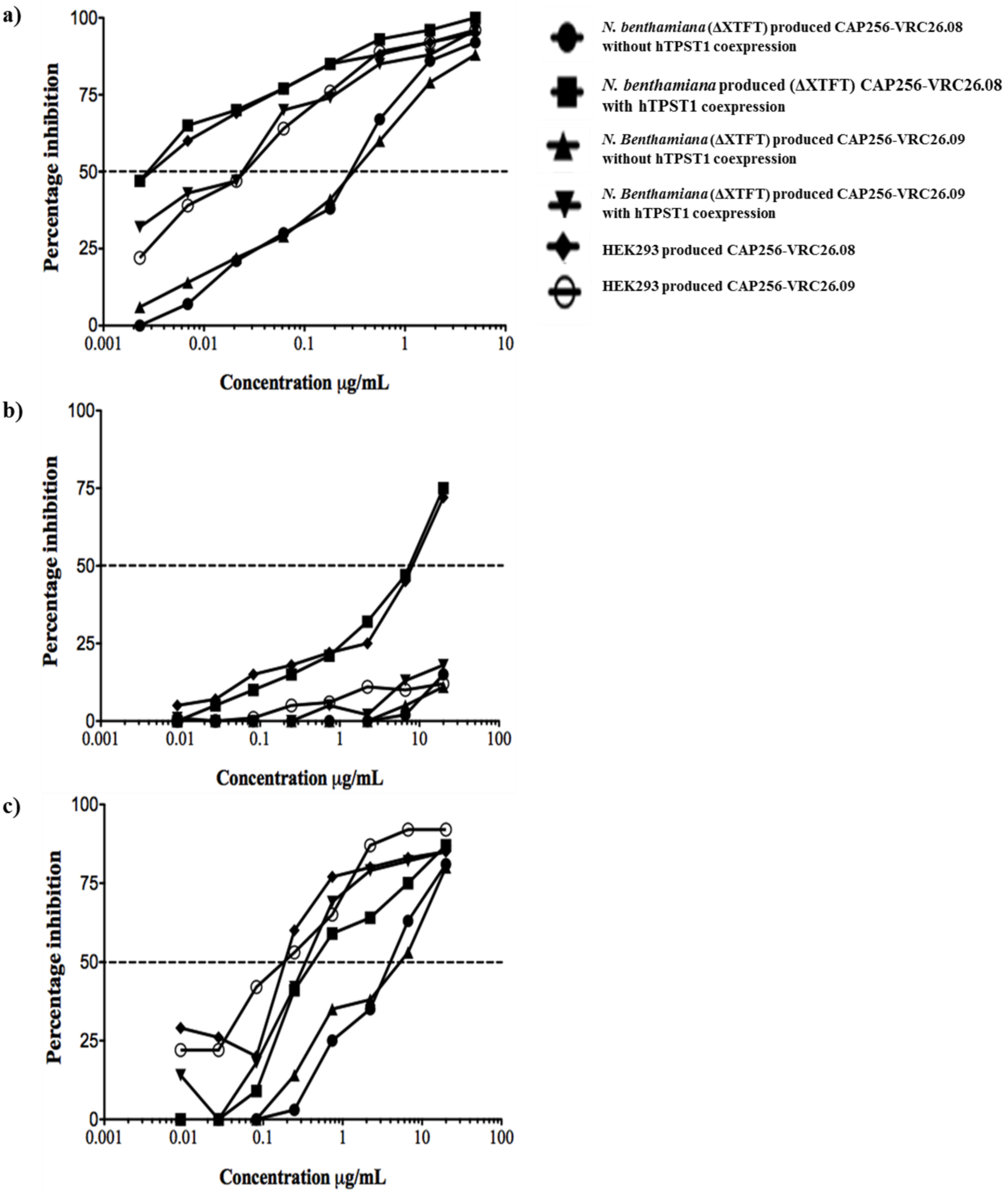

Supplementary Figure S9. Representative inhibition curves for the most sensitive virus for the sutypes C, B and A. Inhibition curves used to calculate the IC_{50} values of (a) the subtype C virus Du422.1, (b) the subtype B PVO.4, and (c) the subtype A Q168.a2 following the TZM-bl cells neutralization assay. Dashed lines are placed at 50% inhibition of HIV-1 infection.
